# A comparative phylogeographic study reveals discordant evolutionary histories of alpine ground beetles (Coleoptera, Carabidae)

**DOI:** 10.1002/ece3.2006

**Published:** 2016-02-26

**Authors:** Yi‐Ming Weng, Man‐Miao Yang, Wen‐Bin Yeh

**Affiliations:** ^1^ Department of EntomologyNational Chung Hsing University250 Kuo‐Kuang RdSouth DistrictTaichungTaiwan40227

**Keywords:** Glacial refugia, *Leistus*, massif de refuge, *Nebria*, nunatak hypothesis

## Abstract

Taiwan, an island with three major mountain ranges, provides an ideal topography to study mountain–island effect on organisms that would be diversified in the isolation areas. Glaciations, however, might drive these organisms to lower elevations, causing gene flow among previously isolated populations. Two hypotheses have been proposed to depict the possible refugia for alpine organisms during glaciations. Nunatak hypothesis suggests that alpine species might have stayed in situ in high mountain areas during glaciations. Massif de refuge, on the other hand, proposes that alpine species might have migrated to lower ice‐free areas. By sampling five sympatric carabid species of *Nebria* and *Leistus*, and using two mitochondrial genes and two nuclear genes, we evaluated the mountain–island effect on alpine carabids and tested the two proposed hypotheses with comparative phylogeographic method. Results from the phylogenetic relationships, network analysis, lineage calibration, and genetic structure indicate that the deep divergence among populations in all *L. smetanai*,* N. formosana*, and *N. niitakana* was subjected to long‐term isolation, a phenomenon in agreement with the nunatak hypothesis. However, genetic admixture among populations of *N. uenoiana* and some populations of *L. nokoensis* complex suggests that gene flow occurred during glaciations, as a massif de refuge depicts. The speciation event in *N. niitakana* is estimated to have occurred before 1.89 million years ago (Mya), while differentiation among isolated populations in *N. niitakana, N. formosana*,* L. smetanai,* and *L. nokoensis* complex might have taken place during 0.65–1.65 Mya. While each of the alpine carabids arriving in Taiwan during different glaciation events acquired its evolutionary history, all of them had confronted the existing mountain ranges.

## Introduction

Mountainous Taiwan was created with continuous arc–continental collision of Eurasian and Philippine Sea plates during late Miocene (Sibuet and Hsu 2004). The drastic uplift of central mountain range (CMR) from north to south was accompanied by the rising of Xueshan and Yushan branch ranges from the northwest and the southwest of CMR, respectively, during 3–1 million years ago (Mya) (Huang et al. 1997; Lee et al. 2006). At present, more than 250 peaks higher than 3000 m distribute in these three mountain ranges. Such discontinuous high mountain areas have been found in North American and Europe to deduce “mountain–island effect,” blocking the dispersal of alpine organisms (Kavanaugh 1985; Tennessen and Zamudio 2008; Schmitt 2009; Schoville et al. 2011, 2012). In addition, alpine environments are extreme and severe, and such harsh characteristics often promote adaptive specializations in insects (Darlington 1943; Byers 1969). For instance, it is easy to find brachypterous carabids with low dispersal capability restricted to isolated mountain ranges in alpine zone (Kano 1930; Habu 1972; Ueno 1989; Ledoux and Roux 1993; Farkac 1995; Zamotajlov and Sciaky 1996; Raupach et al. 2010).

In addition to the “mountain–island effect,” the distribution patterns of alpine organisms were affected by Pleistocene glaciations (Conroy and Cook 2000; DeChaine and Martin 2005b). During ice ages, the decline of sea levels resulted in the land bridge formation between Taiwan and the Asian mainland, which provided opportunities for biota migrating across Taiwan straits (Ray and Adams 2001; Voris 2001; Chang and Chen 2005b; Jang‐Liaw et al. 2008; Huang and Lin 2010; Jang‐Liaw and Chou 2011). The decreasing temperature and dropping mountain snowline (Ono 1988) could make organisms originally living in alpine zones either move to lower elevations (Tsukada 1967; Liew and Chung 2001; DeChaine and Martin 2005a) or find a suitable refugia (Tribsch and Schönswetter 2003; Lohse et al. 2011).

Two common hypotheses for the glacial survival for alpine organisms have been inferred in Alps and north Atlantic area (Schönswetter et al. 2004; Lohse et al. 2011; Schneeweiss and Schoenswetter 2011; Westergaard et al. 2011). Nunatak hypothesis proposes that the alpine species stayed in situ in small ice‐free areas around mountaintops during glacial periods (Dahl 1987). Alternatively, massif de refuge hypothesis suggests that the periphery of mountain ranges provided large refugia for alpine species, allowing the refugees to re‐occupy alpine zones after the retreat of glaciers (Nordal 1987). The scenario that alpine organisms moved down to lower elevation as a response to glaciations has been argued because the brachypterous carabids would have inefficient migration ability to change habitat, or the environments of lower elevation areas might not be suitable for alpine species (Lohse et al. 2011). For the cases in Taiwan, however, many animals migrated from Asian mainland and Japan through the land bridges have clearly shown the possibility for organisms from high latitude and/or high elevation to adapt to and survive at the areas of low elevation of this island (Ota 1998; Creer et al. 2001; Oshida et al. 2006; Jang‐Liaw et al. 2008; Tsai et al. 2014). The repeated glaciation events during Pleistocene would have affected the migratory routes and increased the complexity upon population differentiation of the alpine adaptive organisms. In this study, multiple genetic markers within multiple sympatric species were employed to reconstruct the most likely scenario of Taiwanese alpine animals. Based on the assumption, a deep monophyly observed for carabid populations from different mountain ranges would be compatible with the nunatak hypothesis; and similar genetic compositions found among these populations would be in agreement with the massif de refuge hypothesis.

Ground beetles, tribe Nebriini belonging to the family Carabidae, include two genera *Leistus* and *Nebria* in Taiwan (Terada 2006). Most of them are brachypterous inhabiting alpine zone >3000 m in elevation (Kano 1930; Minowa 1932; Habu 1972; Farkac 1995). These alpine carabids are all endemic, and some of them, for example, the brachypterous species *Nebria niitakana* Kano and *Nebria formosana* Habu distributing in different mountain ranges, are morphologically variable (Habu 1972). *Leistus smetanai* Farkac in Xueshan and Hehuanshan of an altitude of 3100–3400 m is also brachypterous. Yet, *Nebria uenoiana* Habu found usually at an altitude of 2300–3300 m is macropterous. *Leistus nokoensis* Minowa firstly recorded in central CMR and found in all three mountain ranges exhibits recognizable morphological variations. One group of *L. nokoensis* distributes in these mountain ranges between 3000 and 3400 m, while the other group is localized in elevation of 2500 m within the Xueshan range. Thus, these morphologically differentiated populations will be referred as *Leistus nokoensis* complex in this study, until an appropriate taxonomic treatment is available.

More than 50 studies have addressed the population differentiation and phylogeographical pattern of Taiwanese biota, but few of these biota come from alpine zone and none of these studies deals with the mountain–island effect (Wang et al. 2000, 2004; Chang and Chen 2005a; Chiang et al. 2010; Liu et al. 2011; Oshida et al. 2011; Jean et al. 2014). The carabid taxa restricted to alpine environment with low dispersal capability should have experienced similar geological events of orogenesis and periodical glaciations. Therefore, comparative phylogeography, a practical process to investigate the phylogeographical histories of codistributed organisms (Bermingham and Moritz 1998; Arbogast and Kenagy 2001), would be helpful to reconstruct the mountain–island divergent history of these alpine carabids and to address the influences of Quaternary repeated glaciations to their population structure. The aforementioned two hypotheses are tested with phylogenetic relationships, network analysis, lineage calibration, and genetic structure of these alpine carabids.

## Materials and Methods

### Carabids sampling

**Figure 1:**
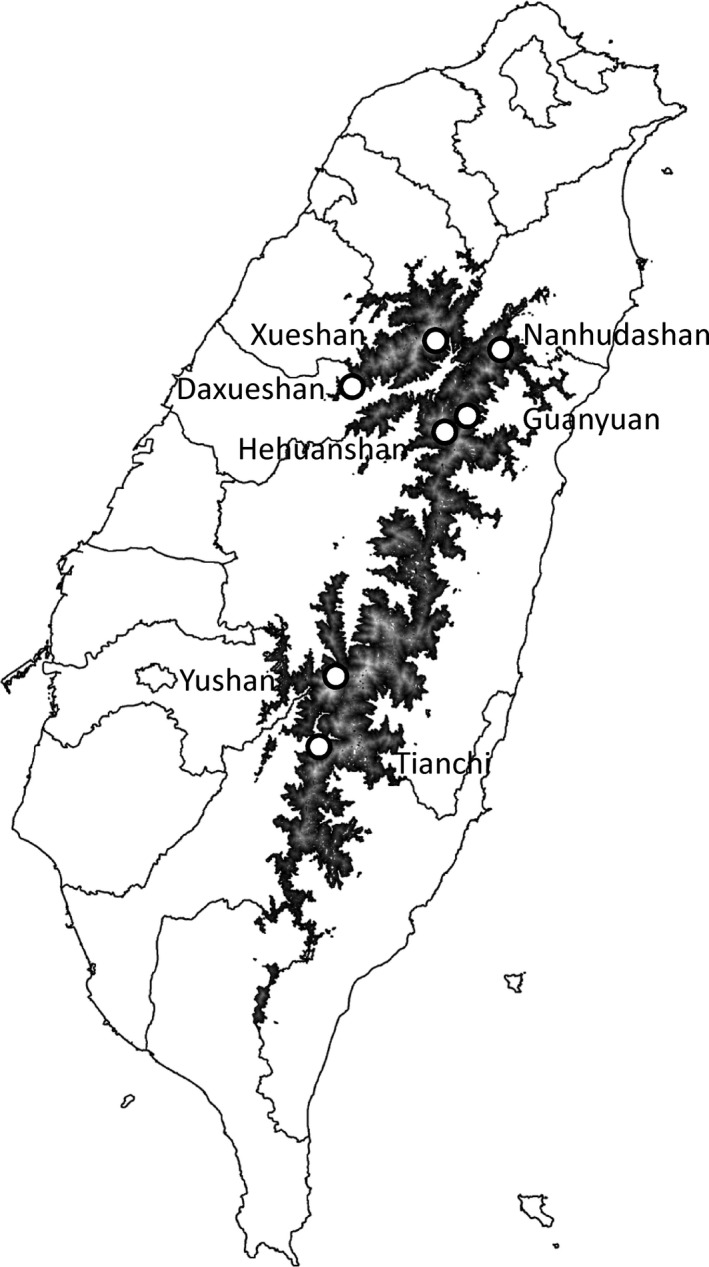
Sampling locations of alpine carabids. Xueshan and Daxueshan are in Xueshan range; Nanhudashan, Hehuanshan, Guanyuan, and Tianchi are in central mountain range; Yushan is in Yushan range. Area in elevation above 2000 m is shaded.

One hundred and thirty‐seven carabids of five alpine *Nebria* and *Leistus* species, that is, *N. uenoiana*,* N. niitakana*,* N. formosana*,* L. smetanai*, and *L. nokoensis* complex, were collected, via pitfall trap or hand capture, from mountain peaks, elevation higher than 2000 m, in ranges of CMR, Xueshan, and Yushan (Fig. 1, Table S1). Sister species of *N. formosana*,* N. niitakana*, and *L. smetanai*, that is, *N*. *reflexa*,* N. ohdaiensis/N. chinensis,* and *L. taiwanensis*, were used as outgroups in phylogenetic tree reconstruction, network analyses, and molecular dating (Habu 1972).

### DNA extraction, amplification and sequencing

Carabids collected were stored in 95% ethanol at −20°C for DNA extraction. Genomic DNA was extracted from metatarsus or metatibia muscle of each ground beetle specimens using the BuccalAmp^™^ DNA Extraction Kit (Epicentre Biotechnologies, Madison, WI). The tissue was ground in 50 *μ*L QuickExtract solution and then centrifuged for 15 sec before incubation at 65°C for 10 min. Following 15‐sec centrifugation, the sample was transferred to 98°C for 2 min. The resultant genomic DNA was stored at −20°C.

Primer pairs used to amplify the mitochondrial COI and 16S rDNA and the nuclear 28S rDNA and *wingless* are listed in Table S2. The PCR assay was performed in a volume of 25 *μ*L containing 2 *μ*L genomic DNA extract, 2.5 *μ*L 10× Taq buffer, 0.5 *μ*L Prime Taq DNA polymerase (GENET BIO, Korea), 0.4 *μ*L dNTP (25 *μ*mol/L), and 1 *μ*L of each primer (10 *μ*mol/L). PCR programming conditions were 94°C for 2 min as the first denaturation followed by 35 cycles of 94°C for 30 sec, 48–55°C for 30 sec, and 72°C for 1 min, with a final extension at 72°C for 10 min. The PCR product was purified with 1% agarose gel using QIAquick Gel Extraction Kit (Qiagen, Hilden, Germany). The resulting DNA product was sequenced in both strands using Taq dye terminator cycle sequencing kit (Applied Biosystems, Foster, CA) and an ABI 377A sequencer. Sequences of COI, 16S rDNA, *wingless*, and 28S rDNA for these carabids have been deposited in GenBank. The accession numbers for COI, *wingless*, 16S rDNA, and 28S rDNA are KT306037‐KT306186, KT306187‐KT306330, KT306331‐KT306481, and KT306482‐KT306548, respectively. The sequences of two outgroup species, that is, *N. chinensis* and *N. ohdaiensis*, were provided by Dave Kavanaugh (Table S4).

### Sequence alignment and the best substitution model

Sequences were aligned and edited using BioEdit 7.0 software (Hall 1999). The best‐fit evolutionary models were inferred in jModelTest 2.1.4 (Guindon and Gascuel 2003; Darriba et al. 2012) using Bayesian information criterion (BIC) (Table S3). A similar model for these genes was applied in lineage calibration using software BEAST (Drummond et al. 2012) (Table S3).

### Analysis of molecular variance (AMOVA) and statistical parsimony network

Nucleotide and haplotype diversity, fixation indexes (*F*_ST_), neutrality test of Tajima's *D* and Fu's *F*_*s*_, and the variance component of each genes were performed in Arlequin ver. 3.5 (Excoffier et al. 2005). Besides, haplotype network was analyzed in genes of COI, 16S rDNA, *wingless* gene, and 28S rDNA using TCS v.1.21 with a 95% connection limitation (Clement et al. 2000).

### Phylogenetic inferences

Phylogenetic trees for each species or species complex were inferred through the maximum‐likelihood (ML), maximum‐parsimony (MP), and Bayesian inference (BI) methods. ML trees were drawn using RAxML ver. 7.0.3 with substitution model of GTR+I+G (Stamatakis 2006). MP trees were conducted using the software TNT (Goloboff et al. 2008), in which the heuristic algorithm by stepwise addition and tree bisection and reconnection (TBR) via parsimony ratchet were performed. One thousand bootstrap resamplings were applied to the ML and MP inferences. BI was performed on software MrBayes 3.1.2 (Huelsenbeck and Ronquist 2001). Generation numbers of metropolis‐coupled Markov chain Monte Carlo (MCMCMC) were set depending on the average standard of split frequencies as below 0.01. For *L. nokoensis* complex, *N. niitakana*, and *N. uenoiana*, there were 2 million generations, and 1 million generations for *L. smetanai* and *N. formosana*. One tree was recorded per 5000 generations and 25 percent of the sampling trees were burnin.

### Lineage calibration and extended Bayesian skyline plot

Divergent time for lineages was evaluated with a strict molecular clock using software BEAST v1.8.0 (Drummond et al. 2012). Substitution rates, that is, 1.17% per lineage in million years (My) for COI and 0.54%/lineage/My for 16S rDNA, suggested optimally for tenebrionid beetles, were used (Papadopoulou et al. 2010; Schoville et al. 2012). Markov chain Monte Carlo (MCMC) sampling was run for each species in 2 × 10^9^ to 8 × 10^9^ generations and sampled every 2 × 10^5^ to 8 × 10^5^ generations, depending on the effective sample size (ESS) values of the estimated parameter traced with software Tracer v1.5 for each carabid species (Rambaut and Drummond 2009). The trees were recorded per 20,000 generations and 10% of the recorded trees were burnin.

A coalescent‐based extended Bayesian skyline plot (EBSP) based on COI and 16S rDNA was generated to reconstruct the population dynamics of each alpine carabid independently with BEAST v1.8.0 (Drummond et al. 2012). The best‐fit models for COI and 16S rDNA of each carabid are shown in Table S3. The MCMC sampling was run for 2 × 10^9^ to 8 × 10^9^ generations and sampled every 2 × 10^5^ to 8 × 10^5^ generations depending on the optimal condition of the estimated parameters. Population demographic expansion was examined with a population growth–decline model implemented in Arlequin 3.5 (Excoffier et al. 2005) by calculating 1000 bootstrap replications for the sum of square deviations (SSD) and the Harpending's Raggedness index (HRi).

## Results

Generally, gene diversities of the populations within each carabid species in Taiwan are larger in COI than in 16S rDNA and *wingless*, except for *N. uenoiana* (Table 1). The Nanhudashan population of *L. smetanai* and Hehuanshan population of *N. formosana* have shown the highest haplotype and nucleotide diversity in *wingless* gene, and yet, high diversities in *wingless* and low in both mitochondrial DNA genes are observed for *N. uenoiana*. Limited sequence variation has been found in 28S rDNA within each carabid species.

**Table 1:** Gene diversity and neutrality test of each carabid population (asterisk means the value statistically significant)

Species	Population	Genes	N^a^	Nh^a^	Hd^a^	100 × *π*	Tajima's D	Fu's Fs
*Leistus. nokoensis*	Xueshan	COI	12	7	0.86	0.53	1.15	−1.02
Complex		16S	11	1	0.00	0.00	0.00	–
		Wingless	11	1	0.00	0.00	0.00	–
	Hehuanshan	COI	13	6	0.64	1.03	−0.27	2.14
		16S	11	2	0.33	0.06	−0.10	0.36
		Wingless	10	2	0.47	0.11	0.82	0.82
	Nanhudashan	COI	4	2	0.50	0.23	−0.75	1.71
		16S	4	1	0.00	0.00	0.00	–
		Wingless	4	1	0.00	0.00	0.00	–
	Daxueshan	COI	5	4	0.90	0.22	−0.17	−1.65*
		16S	5	1	0.00	0.00	0.00	–
		Wingless	5	2	0.60	0.14	1.22	0.63
	Yushan	COI	12	11	0.98	1.01	−0.08	−4.40*
		16S	12	2	0.17	0.03	−1.14	−0.48
		Wingless	12	2	0.30	0.07	−0.19	0.30
*Leistus. smetanai*	Xueshan	COI	5	4	0.90	0.47	−0.75	−0.33
		16S	5	1	0.00	0.00	0.00	–
		Wingless	4	1	0.00	0.00	0.00	–
	Nanhudashan	COI	3	2	0.67	0.31	0.00	1.61
		16S	3	2	0.67	0.26	0.00	1.06
		Wingless	3	3	1.00	0.48	0.00	−0.69
*Nebria. formosana*	Xueshan	COI	10	4	0.53	0.12	−1.67*	−1.34*
		16S	10	1	0.00	0.00	0.00	–
		Wingless	10	1	0.00	0.00	0.00	–
	Hehuanshan	COI	7	2	0.29	0.04	−1.01	−0.09
		16S	10	1	0.00	0.00	0.00	–
		Wingless	6	2	0.53	0.38	1.12	2.50
	Yushan	COI	10	3	0.51	0.09	−0.69	−0.59
		16S	10	2	0.20	0.04	−1.11	−0.34
		Wingless	10	2	0.20	0.05	−1.11	−0.34
*Nebria. niitakana*	Xueshan	COI	8	4	0.79	0.17	−0.30	−1.10
		16S	14	1	0.00	0.00	0.00	–
		Wingless	13	4	0.62	0.23	−0.06	−0.63
	Nanhudashan	COI	14	12	0.98	0.66	0.13	−6.46*
		16S	15	2	0.13	0.03	−1.16	−0.65
		Wingless	15	7	0.78	0.40	0.96	−2.60*
*Nebria. uenoiana*	Xueshan	COI	2	2	1.00	0.47	0.00	1.10
		16S	1	1	–	–	–	–
		Wingless	1	1	–	–	–	–
	Hehuanshan	COI	12	6	0.82	0.30	−0.21	
		16S	12	1	0.00	0.00	0.00	–
		Wingless	11	4	0.80	0.31	0.95	−0.17
	Guanyuan	COI	9	4	0.80	0.30	0.24	0.33
		16S	9	1	0.00	0.00	0.00	–
		Wingless	9	6	0.89	0.48	1.15	−2.33*
	Nanhudashan	COI	9	2	0.22	0.10	−1.51	1.32
		16S	9	1	0.00	0.00	0.00	–
		Wingless	9	4	0.69	0.25	−0.36	−1.04
	Yushan	COI	10	4	0.64	0.31	−0.77	0.62
		16S	10	1	0.00	0.00	0.00	–
		Wingless	7	5	0.90	0.55	0.13	−1.45
	Tianchi	COI	2	1	0.00	0.00	0.00	–
		16S	3	2	0.67	0.12	0.00	0.20
		Wingless	3	2	0.67	0.33	0.00	1.06

a N, number of individuals; Nh, number of haplotypes; Hd, haplotype diversity. Significant values are indicated with an asterisk

### Genetic differentiation and variance component within/among populations

Substantial genetic differentiation among populations of *L. smetanai*,* N. niitakana*, and *N. formosana,* with *F*_ST_ values larger than 0.69, obviously resulted from the mountain–island effect (Table S5). With most *F*_ST_ values <0.3, the differentiation among populations of *N. uenoiana* and some populations of *L. nokoensis* is weak to mild, while high *F*_ST_ values could be found between the southern Yushan population and the other *L. nokoensis* populations (Table S5).

Species of *N. niitakana*,* N. formosana*,* L. smetanai*, and *L. nokoensis* complex have high sequence variation among populations: on average of 88%, 92%, 79%, and 95% for COI, 16S rDNA, *wingless*, and 28S rDNA, respectively (Table 2). Variation component within population is high for *wingless* (83%) and COI (61%) genes, but low for 16S rDNA (13%) in *N. uenoiana*.

**Table 2:** Sequence variation component in each gene of each carabid taxon

Percentage	Components	COI	16S rDNA	*Wingless*	28S rDNA
*Leistus. nokoensis* complex	Among populations	0.78	0.94	0.85	0.88
	Within populations	0.22	0.06	0.15	0.12
*Leistus. smetanai*	Among populations	0.86	0.80	0.75	1.00
	Within populations	0.14	0.20	0.25	0.00
*Nebria. niitakana*	Among populations	0.91	0.98	0.69	1.00
	Within populations	0.09	0.02	0.31	0.00
*Nebria. formosana*	Among populations	0.98	0.98	0.85	0.92
	Within populations	0.02	0.02	0.15	0.08
*Nebria. uenoiana*	Among populations	0.39	0.87	0.17	–a
	Within populations	0.61	0.13	0.83	–a

a No variation was obtain.

Neutrality test shows negative, yet insignificant, Tajima's *D* and/or Fu's *Fs* values for most populations (Table 1), while positive values are found in several populations, such as Hehuanshan of *L. nokoensis* complex. Both Tajima's *D* and Fu's *Fs* test in Xueshan population of *N. formosana* are significantly negative. Fu's *Fs* test for COI of three populations, that is, Daxueshan and Yushan of *L. nokoensis* complex and Xueshan of *N. formosana*, is significantly negative, and similar data have been obtained for Guanyuan of *N. uenoiana* in *wingless* and Nanhudashan of *N. niitakana* in COI and *wingless*.

### Phylogenetic inferences

**Figure 2:**
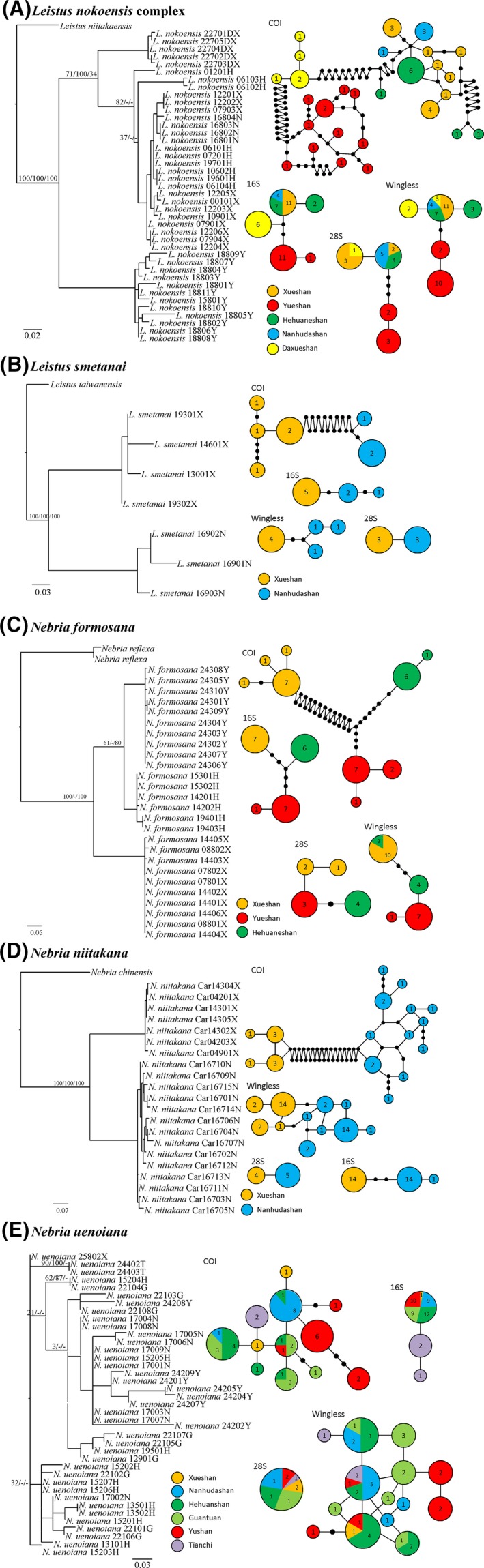
Phylogenetic tree and network analysis of each carabid species; (A) *Leistus nokoensis* complex; (B) *L. smetanai*; (C) *Nebria formosana*; (D) *N. niitakana*; and (E) *N. uenoiana*. Topology is shown in Bayesian inference with maximum‐parsimony and maximum‐likelihood bootstrap values (>50%) on nods.

Phylogenetic inferences based on COI, 16S rDNA, and *wingless* of each species have shown consistent topology in MP, ML, and BI methods. The phylograms have revealed that *L. smetanai*,* N. formosana*, and *N. niitakana* (Fig. 2) from each mountain range have their distinct lineages, while *N. uenoiana,* with distribution over a wider elevation range, has close relationship with each other across all mountain ranges. On the other hand, *L. nokoensis* complex contains both distinct and mixed lineages, although it has similar distribution elevation as *N. uenoiana* (Fig. 2A). Samples from Xueshan, Hehuanshan, and Nanhudashan, which are mixed with each other, together with their sibling lineage of Daxueshan population, form the northern group, to which the southern Yushan population has deep divergence.

### Statistical parsimony network analysis

Parsimony network analysis of mitochondrial and nuclear genes in *L. nokoensis* complex (Fig. 2A) reveals that individuals from the southern Yushan population form a group distinct from the northern group, which consists of Daxueshan, Xueshan, Hehuanshan, and Nanhudashan populations. While Hehuanshan population of the northern group contains the most diverse haplotypes for all three genes, Nanhudashan population is the least divergent in this group. Mitochondrial genes of Daxueshan populations appear to be intermediate between the other northern populations and southern Yushan population. Networks for *L. smetanai* and *N. niitakana* show a deep divergence between Xueshan and Nanhudashan populations (Fig. 2B,D). Parsimony network analysis of COI gene suggests a close relationship between Hehuanshan and Yushan populations in *N. formosana* (Fig. 2C); however, the shared haplotype in *wingless* gene shows a possible close affinity between Hehuanshan and Xueshan populations. *Nebria uenoiana* is the least differentiated species, although the southern Yushan and Tianchi populations have several peculiar haplotypes (Fig. 2E).

### Lineages calibration

**Figure 3:**
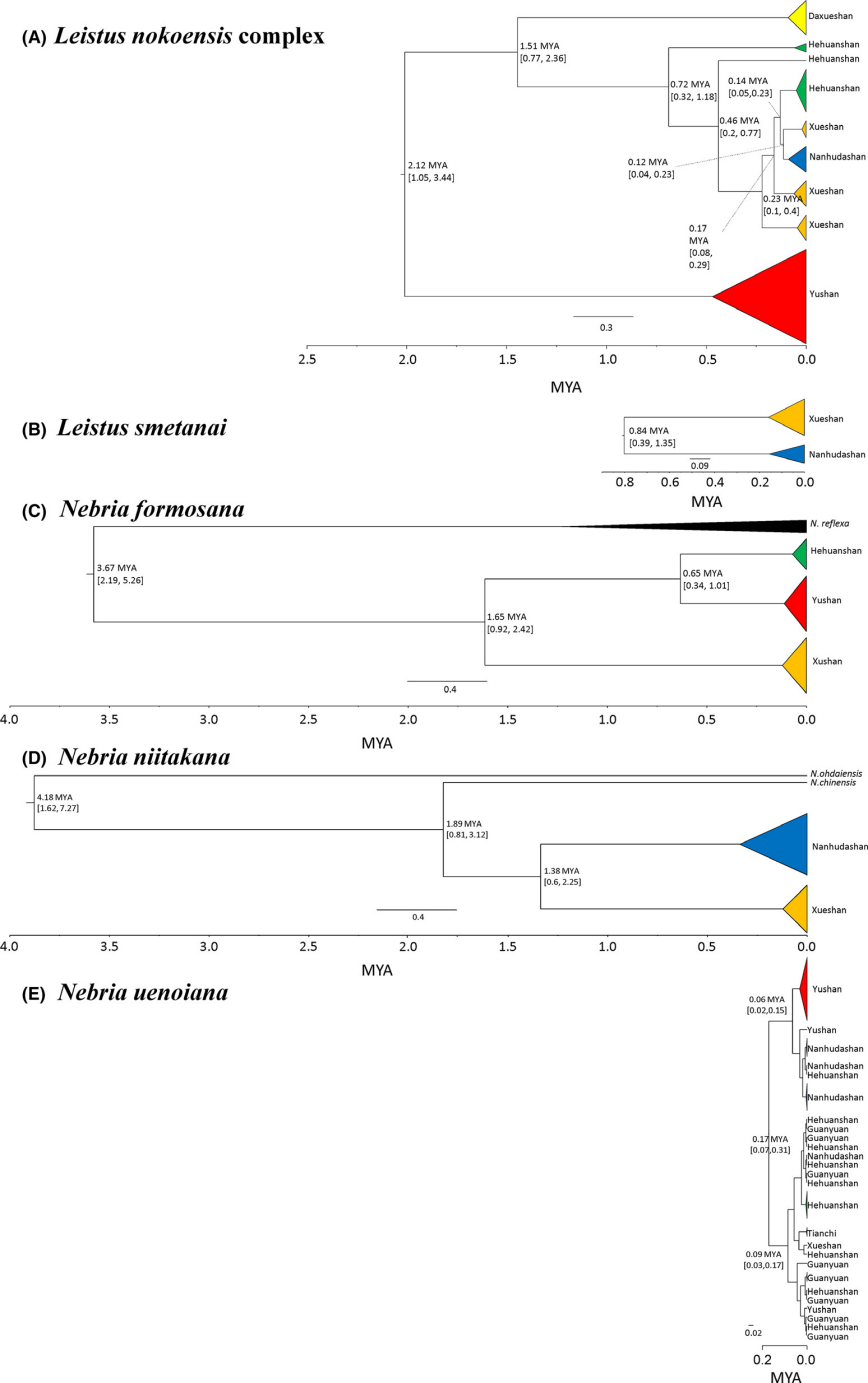
Strict molecular clock calculation based on mitochondrial genes of COI and 16S rDNA for each carabid species. Inferences were performed for each species independently and arranged based on the same timescale.

According to outgroup comparison, the speciation events on *N. formosana* and *N. niitakana* are estimated to have taken place before 3.67 and 1.89 Mya, respectively (Fig. 3). In *L. nokoensis* complex, the split between Yushan and the northern group that occurred around 2.12 Mya suggests that the morphologically recogniza‐ble Yushan population might be a separate species. In *N. formosana*, the divergent one is the northern lineage (Xueshan), while it is the southern lineage (Yushan) in *L. nokoensis* complex.

Differentiation events among isolated populations of *N. formosana*,* N. niitakana*, the northern *L. nokoensis*, and *L. smetanai* took place mainly during 1.65–0.65 Mya. For example, the divergence time between Xueshan and Nanhudashan populations of *L. smetana*i is 0.84 Mya and that of *N. niitakana* is 1.38 Mya. For *N. formosana*, the first divergence between Xueshan and Hehuanshan‐Yushan lineages occurred at 1.65 Mya and that between Hehuanshan and Yushan populations occurred at 0.65 Mya. For *L. nokoensis* complex, the diversified events within northern group occurred <1.51 Mya and the major diversification among populations of Henhuanshan, Nanhudashan, and Xueshan took place between 0.12 and 0.72 Mya. Multiple shallow lineages are observed for *N. uenoiana* and the earliest divergence occurred at ca. 0.17 Mya. Obviously, each carabid species must have had its own evolutionary history.

### Extended Bayesian skyline plot

**Figure 4:**
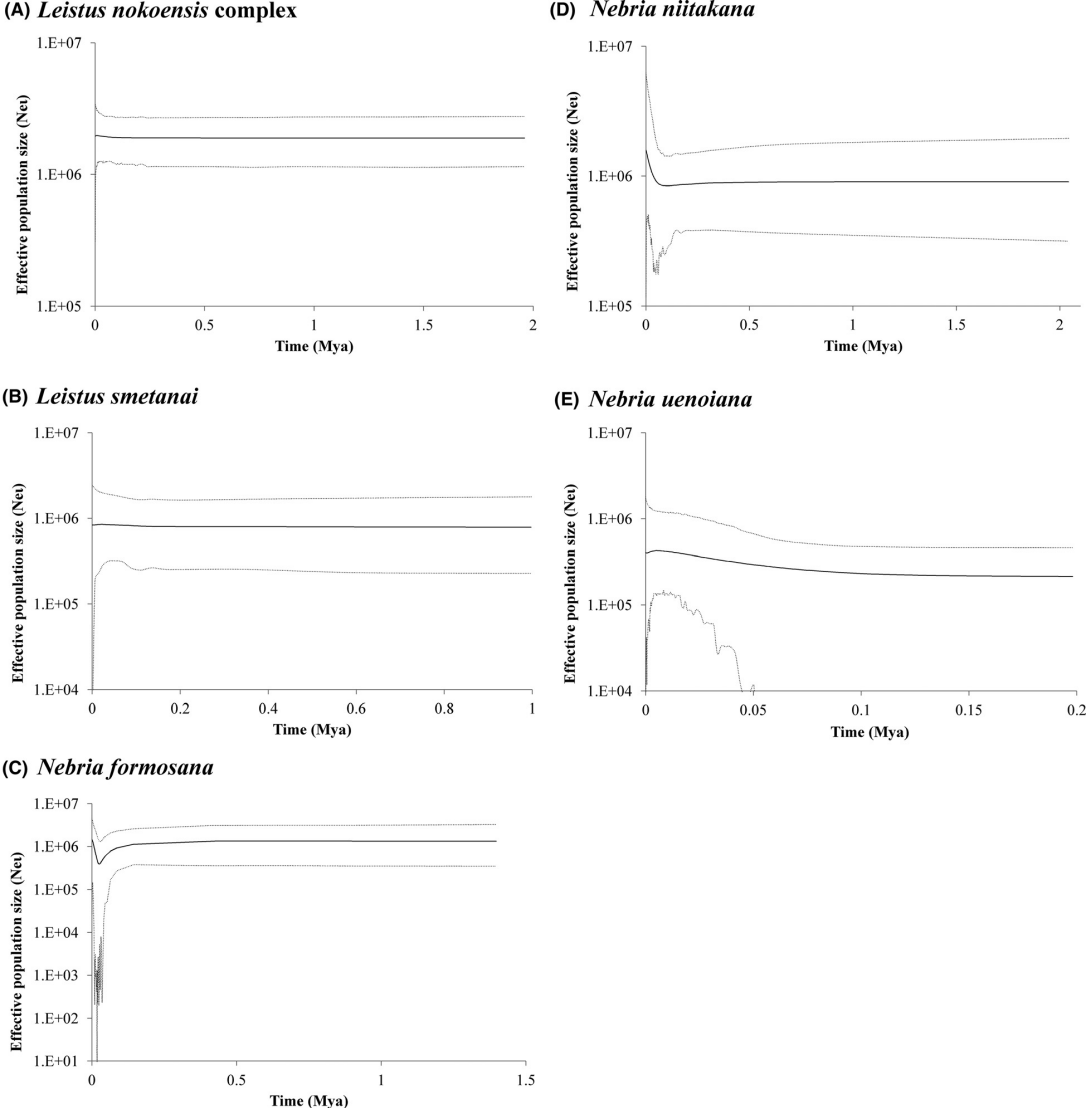
Coalescent‐based extended Bayesian skyline plot of each carabid species. (A) *Leistus nokoensis* complex; (B) *L. smetanai*; (C) *Nebria formosana*; (D) *N. niitakana*; and (E) *N. uenoiana*.

Coalescent‐based extended Bayesian skyline plot shows that these alpine carabids have different demographic patterns, although they had met the same periodical glaciation events in Taiwan. The population size of *Leistus* has remained relatively constant in comparison with *Nebria* during the past 1 million years (Fig. 4). While *N. formosana*,* N. niitakana,* and *N. uenoiana* all have had a recent population growth, *N. formosana* might have confronted with a bottleneck effect in the last glacial maximum (Fig. 4C–E).

## Discussions

Previous studies have shown that several factors, such as glacial events and mountain–island isolations, promoted the diversity of endemic species on Taiwan Island (Huang and Lin 2010; Liu et al. 2011; Oshida et al. 2011; Tsai et al. 2014). The taxa that dwell at alpine areas in mountainous Taiwan have had particular association with the periodical glaciation events which brought biota from high‐latitude regions to this island. Several comparative phylogeographical studies have proposed that organisms inhabiting in the same geological areas would have acquired a similar differentiation history (Arbogast and Kenagy 2001). All alpine carabids were once thought to have gone through the periodical glaciation events and orogenesis in Taiwan. However, alpine carabids in this study have displayed different population structures and phylogeographical patterns. While all alpine organisms would have been affected by the existing mountain ranges in Taiwan, we assume that carabids arriving in Taiwan during different glaciation events would have had different evolutionary histories.

### Origin of Taiwan alpine carabids

It is believed that most organisms in Taiwan colonized from eastern mainland Asia, southeastern Asia, and northern Japan, either through currents or via land bridges during ice ages (Ray and Adams 2001; Voris 2001; Chang and Chen 2005b; Jang‐Liaw et al. 2008; Huang and Lin 2010; Jang‐Liaw and Chou 2011). Based on morphological characters, *N. formosana* and *N. niitakana,* respectively, are sister species of *N. reflexa* and *N. ohdaiensis* from Japan (Habu 1972). However, phylogenetic relationships and molecular dating in this study show that the most recent common ancestor of *N. niitakana* is *N. chinensis* from China tracing back 1.89 Mya, and *N. ohdaiensis* had diverged from *N. niitakana*–*N. chinensis* lineage around 4.18 Mya (Fig. 3D). The split time of *N. formosana* from Japanese species *N. reflexa* is ca. 3.67 Mya, which is much earlier than that for most cases of the peripatric speciation events in Taiwan, that is, 1.5–2.5 Mya (Huang and Lin 2010; Jang‐Liaw and Chou 2011; Tsai et al. 2014).

Although with no outgroup involved, lineage calibration shows the origin of *L. smetanai* and *N. uenoiana* in Taiwan would be earlier than 0.84 and 0.17 Mya, respectively. More than forty individuals of *N. uenoiana* sampled from six locations in three mountain ranges were traced back to ca. 0.17 Mya, revealing the possibility of recent colonization or local differentiation (Fig. 3E).

### Mountain–island effect and population structure of alpine carabids

In *L. nokoensis* complex, the southern Yushan population clearly separated from four northern populations around 2.12 Mya (Fig. 3A). Among the latter populations, the Daxueshan population with great divergence in COI gene appears to have been isolated from the other three, that is, Xueshan, Nanhudashan, and Hehuanshan. In the meantime, morphological variations in body size and color also suggest the existence of mountain–island effect among populations in this species complex (Weng et al. unpublished data). A similar mountain‐isolated pattern might have occurred in *N. formosana*. Resolutions of phylogenetic inferences, network analysis, and variance component analysis have shown that the Xueshan population is clearly isolated from the other two populations, that is, Yushan and Hehuanshan, 1.65 Mya (Fig. 3C). Yet, deep mitochondrial divergence also indicates a possible differentiation in the latter two populations and a probable incomplete lineage sorting might be possible as seen in *wingless* gene of a few individuals from Hehuanshan sharing the haplotype with Xueshan since the other nuclear gene of 28S rDNA without haplotype sharing existence. Each of *L. smetanai* and *N. niitakana* clearly consists of two mountain‐isolated populations although the differentiated patterns are different (Fig. 2B,D). The currently isolated *L. smetanai* populations could be tracked to 0.84 Mya and, for *N. niitakana*, 1.38 Mya. *Nebria uenoiana* adapted to an elevation from 2300 to 3000 m is the only species in this study with well‐developed wings, which might have facilitated the gene flow among populations from the three mountain ranges. Variance components for COI and *wingless* genes are far less among populations than within populations (Table 2). Network analyses also show that several shared haplotypes could have been acquired from different mountain ranges (Fig. 2E). The results of different divergent patterns across carabid taxa in our work are in accordance with the results of a study which shows the two higher dwelling *Nebria ingens* Horn and *Nebria spatulata* Van Dyke present clearly divergent pattern as compared with the broadly distributed *Nebria ovipennis* LeConte that has unclear divergent pattern in Sierra Nevada in California (Schoville et al. 2012).

### The refugia hypotheses

Deep‐divergent patterns among alpine populations in *L. smetanai*,* N. formosana*,* N. niitakana*, and partial *L. nokoensis* complex indicate that they were under long‐term isolation during Pleistocene glacial cycles. The most recent divergent time among these populations was 0.65 Mya in *L. smetanai*. The findings that several glaciations event since then did not trigger genetic exchange among those carabid populations are compatible with the hypothesis of nunatak refugia (Dahl 1987; Knowles 2001; Lohse et al. 2011). In addition, according to the result of EBSP analysis, population size of all four species was either unchanged or decreased during last glacial maximum (LGM), which also likely supports the nunatak refugia for these four carabids (Tsukada 1966; Alley et al. 1997; Ujiié and Ujiié 1999; Hebenstreit et al. 2006).

However, northern populations of *L. nokoensis* complex, that is, Xueshan, Nanhudashan, and Hehuanshan, each forming nonmonophyly, were unlikely processed with the mountain–island effect. Phylogenetic inference shows that Hehuanshan population involves basal and terminal lineages, while Xueshan and Nanhudashan populations merely consist of terminal lineages, implying a possible origin from Hehuanshan. The intensive diversification of Hehuanshan population tends to suggest the possible existence of a refuge in the vicinity for this carabid species, in agreement with massif de refuge hypothesis. Interestingly, network analysis in COI gene that shows no shared haplotype in the three populations implies that the isolation probably had occurred before LGM. Moreover, the fixation index (*F*_ST_ = 0.31) in the informative COI gene between Xueshan and Nanhudashan, being greater than that (ca. 0.10) in Hehuanshan to Xueshan and Nanhudashan (Table S5), indicates that Hehuanshan population might have been the possible origin each for Xueshan and Nanhudashan populations. Taking account into the lower population, identical haplotype was found between Daxueshan and Xueshan populations in two nuclear genes. Since both *wingless* gene and 28S rDNA show similar haplotype network pattern, we infer that the causing of the sharing haplotype is gene flow between populations, which is different from the scenario of *N. formosana*.

*Nebria uenoiana* distributing across a wide elevation range is the only species examined that was not affected by mountain–island effect. Lineage calibration depicting its diversification could be traced to 0.17 Mya. Thus, genetic exchange most likely occurred in the middle elevation or refuge during glaciations, or the gene flow existing among current populations was made possible with the well development of wings of this insect. Moreover, coalescent‐based EBSP data show steady population expansions during LGM and subsequent interglacial contraction (ca. 0.005 Mya).

## Conclusion

In this study, we propose the mountain‐isolation model for four codistributed carabid species in Taiwan, with an additional shallow divergence model for the 5th species *N. uenoiana*. The geographical isolation of alpine‐dwelling carabids from mountain ranges represents a typical mountain–island effect which confers these carabids with different history of differentiation in Taiwan. For *N. formosana*, the divergent one is the northern Xueshan lineage, while it is the southern Yushan lineage in *L. nokoensis* complex. All alpine carabids had confronted the existing mountain ranges, and each of the them arriving in Taiwan during different glaciation events acquired its evolutionary history. While the current comparative phylogeographical data on most carabid species tend to support the Nunatak hypothesis, those on *N. uenoiana* and the northern group of *L. nokoensis* complex, with recent gene flow in populations under certain circumstances, are in agreement with the massif de refuge hypothesis.

## Conflict of Interest

None declared.

## Data Accessibility

DNA sequences: Genbank accession numbers KT306037‐KT306186 for COI gene, KT306187‐KT306330 for *wingless* gene, KT306331‐KT306481 for 16S rDNA, and KT306482‐KT306548 for 28S rDNA.

## Supporting information


**Table S1.** Individuals of each carabid population.
**Table S2.** Forward (F) and reverse (R) PCR primers of each gene for carabids.
**Table S3.** The best‐fit evolutionary models examined from jModelTest.
**Table S4.** Number of sequences for each gene of five carabid species.
**Table S5.** *F*_ST_ values among populations of each *Leistus* and *Nebria* species.
**Table S6.** Proportional divergences among populations of each *Leistus* and *Nebria* species.

## References

[ece32006-bib-0001] Alley, R. B. , P. A. Mayewski , T. Sowers , M. Stuiver , K. C. Taylor , and P. U. Clark . 1997 Holocene climatic instability: a prominent, widespread event 8200 yr ago. Geology 25:483‐486.

[ece32006-bib-0002] Arbogast, B. S. , and G. Kenagy . 2001 Comparative phylogeography as an integrative approach to historical biogeography. J. Biogeogr. 28:819–825.

[ece32006-bib-0003] Bermingham, E. , and C. Moritz . 1998 Comparative phylogeography: concepts and applications. Mol. Ecol. 7:367–369.

[ece32006-bib-0004] Byers, G. W. 1969 Evolution of wing reduction in crane flies (Diptera: Tipulidae). Evolution 23:346–354. 10.1111/j.1558-5646.1969.tb03517.x 28562884

[ece32006-bib-0005] Chang, C. H. , and J. H. Chen . 2005a Taxonomic status and intraspecific phylogeography of two sibling species of Metaphire (Oligochaeta: Megascolecidae) in Taiwan. Pedobiologia 49:591–600.

[ece32006-bib-0006] Chang, C. H. , and J. H. Chen . 2005b Three new species of octothecate pheretimoid earthworms from Taiwan, with discussion on the biogeography of related species. J. Nat. Hist. 39:1469–1482.

[ece32006-bib-0007] Chiang, T. Y. , H. D. Lin , K. T. Shao , and K. C. Hsu . 2010 Multiple factors have shaped the phylogeography of Chinese spiny loach *Cobitis sinensis* in Taiwan as inferred from mitochondrial DNA variation. J. Fish Biol. 76:1173–1189. 20409169 10.1111/j.1095-8649.2010.02589.x

[ece32006-bib-0008] Clement, M. , D. Posada , and K. A. Crandall . 2000 TCS: a computer program to estimate gene genealogies. Mol. Ecol. 9:1657–1659. 11050560 10.1046/j.1365-294x.2000.01020.x

[ece32006-bib-0009] Conroy, C. , and J. Cook . 2000 Phylogeography of a post‐glacial colonizer: *Microtus longicaudus* (Rodentia: Muridae). Mol. Ecol. 9:165–175. 10672160 10.1046/j.1365-294x.2000.00846.x

[ece32006-bib-0010] Creer, S. , A. Malhotra , R. Thorpe , and W. H. Chou . 2001 Multiple causation of phylogeographical pattern as revealed by nested clade analysis of the bamboo viper (*Trimeresurus stejnegeri*) within Taiwan. Mol. Ecol. 10:1967–1981. 11555241 10.1046/j.0962-1083.2001.01332.x

[ece32006-bib-0011] Dahl, E. 1987 The nunatak theory reconsidered. Ecol. Bull. 38:77–94.

[ece32006-bib-0012] Darlington, P. J. 1943 Carabidae of mountains and islands: data on the evolution of isolated faunas, and on atrophy of wings. Ecol. Monogr. 13:37–61.

[ece32006-bib-0013] Darriba, D. , G. L. Taboada , R. Doallo , and D. Posada . 2012 jModelTest 2: more models, new heuristics and parallel computing. Nat. Methods 9:772. 22847109 10.1038/nmeth.2109 PMC4594756

[ece32006-bib-0014] DeChaine, E. G. , and A. P. Martin . 2005a Historical biogeography of two alpine butterflies in the Rocky Mountains: broad‐scale concordance and local‐scale discordance. J. Biogeogr. 32:1943–1956.

[ece32006-bib-0015] DeChaine, E. G. , and A. P. Martin . 2005b Marked genetic divergence among sky island populations of *Sedum lanceolatum* (Crassulaceae) in the Rocky Mountains. Am. J. Bot. 92:477–486. 21652425 10.3732/ajb.92.3.477

[ece32006-bib-0016] Drummond, A. J. , M. A. Suchard , D. Xie , and A. Rambaut . 2012 Bayesian phylogenetics with BEAUti and the BEAST 1.7. Mol. Biol. Evol. 29:1969–1973. 22367748 10.1093/molbev/mss075 PMC3408070

[ece32006-bib-0017] Excoffier, L. , G. Laval , and S. Schneider . 2005 Arlequin (version 3.0): an integrated software package for population genetics data analysis. Evol. Bioinform. Online 1:47–50. 19325852 PMC2658868

[ece32006-bib-0018] Farkac, J. 1995 Sixteen new species of *Leistus* from Asia (Coleóptera: Carabidae: Nebriini). Acta Soc. Zool. Bohem. 59:145–163.

[ece32006-bib-0019] Goloboff, P. A. , J. S. Farris , and K. C. Nixon . 2008 TNT, a free program for phylogenetic analysis. Cladistics 24:774–786.

[ece32006-bib-0020] Guindon, S. , and O. Gascuel . 2003 A simple, fast, and accurate algorithm to estimate large phylogenies by maximum likelihood. Syst. Biol. 52:696–704. 14530136 10.1080/10635150390235520

[ece32006-bib-0021] Habu, A. 1972 Notes and descriptions of Formosan Carabidae taken by Dr. S.‐I. ueno in 1961 (Coleoptera: Carabidae). I. on three *Nebria* species. Trans. Shikoku Entomol. Soc. 11:71–80.

[ece32006-bib-0022] Hall, T. A. 1999 BioEdit: a user‐friendly biological sequence alignment editor and analysis program for Windows 95/98/NT. Nucleic Acids Symp. Ser. 41:95–98.

[ece32006-bib-0023] Hebenstreit, R. , M. Böse , and A. Murray . 2006 Late Pleistocene and early Holocene glaciations in Taiwanese mountains. Quatern. Int. 147:76–88.

[ece32006-bib-0024] Huang, J. P. , and C. P. Lin . 2010 Diversification in subtropical mountains: phylogeography, Pleistocene demographic expansion, and evolution of polyphenic mandibles in Taiwanese stag beetle, *Lucanus formosanus* . Mol. Phylogenet. Evol. 57:1149–1161. 20971199 10.1016/j.ympev.2010.10.012

[ece32006-bib-0025] Huang, C. Y. , W. Y. Wu , C. P. Chang , et al. 1997 Tectonic evolution of accretionary prism in the arc‐continent collision terrane of Taiwan. Tectonophysics 281:31–51.

[ece32006-bib-0026] Huelsenbeck, J. P. , and F. Ronquist . 2001 MRBAYES: Bayesian inference of phylogenetic trees. Bioinformatics 17:754–755. 11524383 10.1093/bioinformatics/17.8.754

[ece32006-bib-0027] Jang‐Liaw, N. H. , and W. H. Chou . 2011 Phylogeography of the fanged dicroglossine frog, *Limnonectes fujianensis* (Anura, Ranidae), in Taiwan. Zoolog. Sci. 28:254–263. 21466342 10.2108/zsj.28.254

[ece32006-bib-0028] Jang‐Liaw, N. H. , T. H. Lee , and W. H. Chou . 2008 Phylogeography of *Sylvirana latouchii* (Anura, Ranidae) in Taiwan. Zoolog. Sci. 25:68–79. 18275248 10.2108/zsj.25.68

[ece32006-bib-0029] Jean, C. T. , C. Y. Wu , K. C. Tsai , et al. 2014 Population genetic structure in the endemic cyprinid fish *Microphysogobio alticorpus* in Taiwan: evidence for a new phylogeographical area. Biochem. Syst. Ecol. 57:108–116.

[ece32006-bib-0030] Kano, T. 1930 Contribution to the Carabideous‐Fauna of Formosa. Trans. Nat. Hist. Soc. Formos. 20:24–31.

[ece32006-bib-0031] Kavanaugh, D. H. (1985) On wing atrophy in carabid beetles (Coleoptera: Carabidae), with special reference to Nearctic *Nebria* Pp. 408–431 *in* Ball G.E., ed. Taxonomy, Phylogeny, and Zoogeography of Beetles and Ants: a Volume Dedicated to the Memory of Philip J. Darlington, Jr. 1904‐1983. Dr. W. Junk Publishers, Dordrecht, Netherlands.

[ece32006-bib-0032] Knowles, L. L. 2001 Did the Pleistocene glaciations promote divergence? Tests of explicit refugial models in montane grasshopprers. Mol. Ecol. 10:691–701. 11298980 10.1046/j.1365-294x.2001.01206.x

[ece32006-bib-0033] Ledoux, G. , and P. Roux . 1993 *Nebria* (*Boreonebria*) *bousqueti*, nouvelle espece de Taiwan (Coleoptera, Nebriidae). Nouvelle Revue d'Entomologie 10:53–54.

[ece32006-bib-0034] Lee, Y. H. , C. C. Chen , T. K. Liu , et al. 2006 Mountain building mechanisms in the Southern Central Range of the Taiwan Orogenic Belt—From accretionary wedge deformation to arc–continental collision. Earth Planet. Sci. Lett. 252:413–422.

[ece32006-bib-0035] Liew, P. , and N. Chung . 2001 Vertical migration of forests during the last glacial period in subtropical Taiwan. Western Pac. Earth Sci. 1:405–414.

[ece32006-bib-0036] Liu, M. Y. , C. S. Tzeng , and H. D. Lin . 2011 Phylogeography and the genetic structure of the land‐locked freshwater prawn *Macrobrachium asperulum* (Crustacea: Decapoda: Palaemonidae) in Taiwan. Hydrobiologia 671:1–12.

[ece32006-bib-0037] Lohse, K. , J. A. Nicholls , and G. N. Stone . 2011 Inferring the colonization of a mountain range—refugia vs. nunatak survival in high alpine ground beetles. Mol. Ecol. 20:394–408. 21073591 10.1111/j.1365-294X.2010.04929.x

[ece32006-bib-0038] Minowa, S. 1932 New and hitherto‐unrecorded Carabidae from Formosa (I). Trans. Nat. Hist. Soc. Formos. 22:281–292.

[ece32006-bib-0039] Nordal, I. 1987 Tabula rasa after all? Botanical evidence for ice‐free refugia in Scandinavia reviewed. J. Biogeogr. 14:377–388.

[ece32006-bib-0040] Ono, Y. 1988 Last glacial snowline altitude and paleoclimate of the eastern Asia. Quat. Res. 26:271–280.

[ece32006-bib-0041] Oshida, T. , J. K. Lee , L. K. Lin , and Y. J. Chen . 2006 Phylogeography of Pallas's squirrel in Taiwan: geographical isolation in an arboreal small mammal. J. Mammal. 87:247–254.

[ece32006-bib-0042] Oshida, T. , L. K. Lin , S. W. Chang , Y. J. Chen , and J. K. Lin . 2011 Phylogeography of two sympatric giant flying squirrel subspecies, *Petaurista alborufus* lena and *P. philippensis* grandis (Rodentia: Sciuridae), in Taiwan. Biol. J. Linn. Soc. 102:404–419.

[ece32006-bib-0043] Ota, H. 1998 Geographic patterns of endemism and speciation in amphibians and reptiles of the Ryukyu Archipelago, Japan, with special reference to their paleogeographical implications. Res. Popul. Ecol. 40:189–204.

[ece32006-bib-0044] Papadopoulou, A. , I. Anastasiou , and A. P. Vogler . 2010 Revisiting the insect mitochondrial molecular clock: the mid‐Aegean trench calibration. Mol. Biol. Evol. 27:1659–1672. 20167609 10.1093/molbev/msq051

[ece32006-bib-0045] Rambaut, A. , and Drummond, A. (2009) Tracer v1. 5. 2009 Available from http://beast. bio. ed. ac. uk. Tracer.

[ece32006-bib-0046] Raupach, M. J. , J. J. Astrin , K. Hannig , et al. 2010 Molecular species identification of Central European ground beetles (Coleoptera: Carabidae) using nuclear rDNA expansion segments and DNA barcodes. Front. Zool. 7:1–15. 20836845 10.1186/1742-9994-7-26 PMC2945340

[ece32006-bib-0047] Ray, N. , and J. M. Adams . 2001 A GIS‐based vegetation map of the world at the last glacial maximum (25,000–15,000 BP). Internet Archaeol. 11:1–44.

[ece32006-bib-0048] Schmitt, T. 2009 Biogeographical and evolutionary importance of the European high mountain systems. Front. Zool. 6:9. 19480666 10.1186/1742-9994-6-9 PMC2700098

[ece32006-bib-0049] Schneeweiss, G. M. , and P. Schoenswetter . 2011 A re‐appraisal of nunatak survival in arctic‐alpine phylogeography. Mol. Ecol. 20:190–192. 21265053 10.1111/j.1365-294x.2010.04927.x

[ece32006-bib-0050] Schönswetter, P. , A. Tribsch , I. Stehlik , and H. Niklfeld . 2004 Glacial history of high alpine *Ranunculus glacialis* (Ranunculaceae) in the European Alps in a comparative phylogeographical context. Biol. J. Linn. Soc. 81:183–195.

[ece32006-bib-0051] Schoville, S. D. , M. Stuckey , and G. K. Roderick . 2011 Pleistocene origin and population history of a neoendemic alpine butterfly. Mol. Ecol. 20:1233–1247. 21244539 10.1111/j.1365-294X.2011.05003.x

[ece32006-bib-0052] Schoville, S. D. , G. K. Roderick , and D. H. Kavanaugh . 2012 Testing the ‘Pleistocene species pump' in alpine habitats: lineage diversification of flightless ground beetles (Coleoptera: Carabidae: *Nebria*) in relation to altitudinal zonation. Biol. J. Linn. Soc. 107:95–111.

[ece32006-bib-0055] Sibuet, J. C. , and S. K. Hsu . 2004 How was Taiwan created? Tectonophysics 379:159–181.

[ece32006-bib-0056] Stamatakis, A. 2006 RAxML‐VI‐HPC: maximum likelihood‐based phylogenetic analyses with thousands of taxa and mixed models. Bioinformatics 22:2688–2690. 16928733 10.1093/bioinformatics/btl446

[ece32006-bib-0057] Tennessen, J. A. , and K. R. Zamudio . 2008 Genetic differentiation among mountain island populations of the striped plateau lizard, *Sceloporus virgatus* (Squamata: Phrynosomatidae). Copeia 2008:558–564.

[ece32006-bib-0058] Terada, K. 2006 A checklist of the Carabidae (Coleoptera) recorded from Taiwan. Misc. Rep. Hiwa Mus. Nat. Hist. 46:1–72.

[ece32006-bib-0059] Tribsch, A. , and P. Schönswetter . 2003 Patterns of endemism and comparative phylogeography confirm palaeoenvironmental evidence for Pleistocene refugia in the Eastern Alps. Taxon 52:477–497.

[ece32006-bib-0060] Tsai, C. L. , X. Wan , and W. B. Yeh . 2014 Differentiation in stag beetles, *Neolucanus swinhoei* complex (Coleoptera: Lucanidae): four major lineages caused by periodical Pleistocene glaciations and separation by a mountain range. Mol. Phylogenet. Evol. 78:245–259. 24837623 10.1016/j.ympev.2014.05.004

[ece32006-bib-0061] Tsukada, M. 1966 Late Pleistocene vegetation and climate in Taiwan (Formosa). Proc. Natl Acad. Sci. USA 55:543–548. 16591341 10.1073/pnas.55.3.543 PMC224184

[ece32006-bib-0062] Tsukada, M. 1967 Vegetation in subtropical Formosa during the Pleistocene glaciations and the Holocene. Palaeogeogr. Palaeoclimatol. Palaeoecol. 3:49–64.

[ece32006-bib-0063] Ueno, S. I. 1989 The Taiwanese species of the genus *Epaphiopsis* (Coleoptera, Trechinae). Bull. Nat. Sci. Mus. Ser. A. Zool. 15:105–137.

[ece32006-bib-0064] Ujiié, H. , and Y. Ujiié . 1999 Late Quaternary course changes of the Kuroshio Current in the Ryukyu Arc region, northwestern Pacific Ocean. Mar. Micropaleontol. 37:23–40.

[ece32006-bib-0065] Voris, H. K. 2001 Maps of Pleistocene sea levels in Southeast Asia: shorelines, river systems and time durations. J. Biogeogr. 27:1153–1167.

[ece32006-bib-0066] Wang, J. P. , K. C. Hsu , and T. Y. Chiang . 2000 Mitochondrial DNA phylogeography of *Acrossocheilus paradoxus* (Cyprinidae) in Taiwan. Mol. Ecol. 9:1483–1494. 11050544 10.1046/j.1365-294x.2000.01023.x

[ece32006-bib-0067] Wang, J. P. , H. D. Lin , S. Huang , et al. 2004 Phylogeography of *Varicorhinus barbatulus* (Cyprinidae) in Taiwan based on nucleotide variation of mtDNA and allozymes. Mol. Phylogenet. Evol. 31:1143–1156. 15120406 10.1016/j.ympev.2003.10.001

[ece32006-bib-0068] Westergaard, K. B. , I. G. Alsos , M. Popp , et al. 2011 Glacial survival may matter after all: nunatak signatures in the rare European populations of two west‐arctic species. Mol. Ecol. 20:376–393. 21156004 10.1111/j.1365-294X.2010.04928.x

[ece32006-bib-0069] Zamotajlov, A. , and R. Sciaky . 1996 Contribution to the knowledge of Patrobinae (Coleoptera, Carabidae) from south‐east Asia. Coleoptera 20:1–63.

