# Supplementary material for: A comparative phylogeographic study reveals discordant evolutionary histories of alpine ground beetles (Coleoptera, Carabidae)
Source: Ecol Evol. 2016 Feb 26;6(7):2061–73. doi: 10.1002/ece3.2006 (PMC4768753; doi:10.1002/ece3.2006)
Supplement: Supplementary file 1 — Table S1. Individuals of each carabid population. Table S2. Forward (F) and reverse (R) PCR primers of each gene for carabids. Table S3. The best‐fit evolutionary models examined from jModelTest. Table S4. Number of sequences for each gene of five carabid species. Table S5. F ST values among populations of each Leistus and Nebria species. Table S6. Proportional divergences among populations of each Leistus and Nebria species. [file ECE3-6-2061-s001.docx]

Table S1 Individuals of each carabid populations

|  |  | *L. nokoensis* | *L. smetanai* | *N. niitakana* | *N. formosana* | *N. uenoiana* |
| --- | --- | --- | --- | --- | --- | --- |
| Xueshan  Range | Xueshan | 12 | 5 | 14 | 10 | 2 |
|  | Daxueshan | 5 |  |  |  |  |
| Central  Mountain  Range | Nanhudashan | 4 | 3 | 15 | - | 9 |
|  | Hehuanshan | 13 | - | - | 7 | 12 |
|  | Guanyuan | - | - | - | - | 9 |
|  | Tainchi | - | - | - | - | 2 |
| Yushan Range | Yushan | 12 | - | - | 10 | 10 |

Table S2 Forward (F) and reverse (R) PCR primers of each gene for carabids

| Gene | Primer name | F/R | Sequence (5'–3') | Reference |
| --- | --- | --- | --- | --- |
| COI | Col46 | F | 5'-AAC CAT AAA GAT ATT GGA AC-3' | (Tsai *et al.* 2014) |
|  | niit-COI-F1 | F | 5'-TTA TTT TTG GRG CAT GAG CAG G-3' | In this study |
|  | Col731 | R | 5'-CAA CAT TTA TTT TGA TTT TTT GG-3' | (Tsai *et al.* 2014) |
| 16S rDNA (16S) | 16SR21 | F | 5'-GCC TGT TTA TCA AAA ACA T-3' | (Yeh *et al.* 2004) |
|  | 16S22 | R | 5'-TGA TCT GAG TTC AGA CCG G-3' | (Yeh *et al.* 2004) |
| wingless (Wg) | Wg1A | F | 5'-GAR TGY AAR TGY CAY GGY ATG TCT GG-3' | (Cryan *et al.* 2001) |
|  | Neb-Wg-F1 | F | 5'-ATG CAA ACC TGT TGG ATG CG-3' | In this study |
|  | Leis-Wg-R1 | R | 5'-CGC ATC CAA CAG GTT TGC AT-3' | In this study |
|  | 3WG2-TG | R | 5'-TGY ACA TTC CAY TGG TGY TGC GAG-3' | (Maddison & Ober 2011) |
|  | Neb-Wg-R1 | R | 5'-GCA CAY CGC TCM ACC ACA ACA AT-3' | In this study |
| 28S rDNA (28S) | 28Se | F | 5'-TCC GTA ACT TCG GAA CAA GGA TT-3' | (Lin *et al.* 2003) |
|  | 28Sf | R | 5'-AGT TTG ACT GGG GCG GTA CA-3' | (Lin *et al.* 2003) |

Table S3 The best-fit evolutionary models examined from jModeltest

| Species | Genes | CCMC* | EBSP* | Bayesian |
| --- | --- | --- | --- | --- |
| *L. smetanai* | COI | HKY | HKY | HKY |
|  | 16S | HKY+I | HKY+I | F81+I |
|  | wingless | - | - | JC69 |
| *L. nokoensis* complex | COI | HKY+I+G | HKY+I+G | HKY+I+G |
|  | 16S | HKY | HKY | F81 |
|  | wingless | - | - | JC69 |
| *N. formosana* | COI | HKY | HKY | HKY |
|  | 16S | HKY | HKY | F81 |
|  | wingless | - | - | JC69 |
| *N. niitakana* | COI | HKY+I | HKY+I | HKY+I |
|  | 16S | HKY | HKY | F81 |
|  | wingless | - | - | HKY+I |
| *N. uenoiana* | COI | HKY+I | HKY | HKY |
|  | 16S | HKY | HKY | F81 |
|  | wingless | - | - | JC69+I |
| *N. formosana* + outgroup | COI | HKY+I | HKY+I | HKY+I |
|  | 16S | HKY+I | HKY+I | HKY+I |
|  | wingless | - | - | K80 |
| *N. niitakana* + outgroup | COI | HKY | HKY+I | HKY+I |
|  | 16S | TN93+I | TN93+I | F81+I |
|  | wingless | - | - | HKY+I |

* CCMC: Coalescent Constant size Molecular Clock; EBSP: Extended Bayesian Skyline Plot

Table S4 Numbers of sequences for each gene of each carabid species

| Species | COI | 16S rDNA | wingless | 28S rDNA |
| --- | --- | --- | --- | --- |
| *L. nokoensis* | 46 | 41 | 41 | 21 |
| *L. smetanai* | 8 | 8 | 7 | 6 |
| *N. formosana* | 27 | 26 | 26 | 10 |
| *N. niitakana* | 22 | 29 | 28 | 9 |
| *N. uenoiana* | 44 | 44 | 40 | 18 |

|  | Genes | X/N | X/H | X/Y | N/H | H/Y | N/Y | DX/X* G/X* | DX/H* G/H* | DX/N* G/N* | DX/Y*  G/Y* | T/G | X/T | H/T | N/T | Y/T |
| --- | --- | --- | --- | --- | --- | --- | --- | --- | --- | --- | --- | --- | --- | --- | --- | --- |
| *L. nokoensis* complex | COI | 0.31 | 0.10 | 0.84 | 0.13 | 0.77 | 0.84 | 0.88 | 0.76 | 0.94 | 0.81 | - | - | - | - | - |
|  | 16S | 0.00 | 0.16 | 0.97 | -0.01 | 0.92 | 0.96 | 1.00 | 0.87 | 1.00 | 0.96 | - | - | - | - | - |
|  | Wingless | 0.00 | 0.24 | 0.94 | 0.08 | 0.88 | 0.92 | 0.44 | 0.26 | 0.19 | 0.88 | - | - | - | - | - |
|  | 28S | 0.45 | 0.50 | 0.88 | 0.00 | 0.93 | 0.93 | -0.02 | 1.00 | 1.00 | 0.92 | - | - | - | - | - |
| *L. smetanai* | COI | 0.86 | - | - | - | - | - | - | - | - | - | - | - | - | - | - |
|  | 16S | 0.80 | - | - | - | - | - | - | - | - | - | - | - | - | - | - |
|  | Wingless | 0.75 | - | - | - | - | - | - | - | - | - | - | - | - | - | - |
|  | 28S | 1.00 | - | - | - | - | - | - | - | - | - | - | - | - | - | - |
| *N. niitakana* | COI | 0.91 | - | - | - | - | - | - | - | - | - | - | - | - | - | - |
|  | 16S | 0.98 | - | - | - | - | - | - | - | - | - | - | - | - | - | - |
|  | Wingless | 0.69 | - | - | - | - | - | - | - | - | - | - | - | - | - | - |
|  | 28S | 1.00 | - | - | - | - | - | - | - | - | - | - | - | - | - | - |
| *N. formosana* | COI | - | 0.98 | 0.98 | - | 0.96 | - | - | - | - | - | - | - | - | - | - |
|  | 16S | - | 1.00 | 0.98 | - | 0.97 | - | - | - | - | - | - | - | - | - | - |
|  | Wingless | - | 0.69 | 0.98 | - | 0.64 | - | - | - | - | - | - | - | - | - | - |
|  | 28S | - | 0.93 | 0.80 | - | 1.00 | - | - | - | - | - | - | - | - | - | - |
| *N. uenoiana* | COI | 0.23 | 0.20 | 0.22 | 0.50 | 0.45 | 0.31 | 0.07 | 0.14 | 0.40 | 0.45 | 0.40 | 0.40 | 0.40 | 0.80 | 0.59 |
|  | 16S | 0.00 | 0.00 | 0.00 | 0.00 | 0.00 | 0.00 | 0.00 | 0.00 | 0.00 | 0.00 | 0.91 | 0.60 | 0.93 | 0.96 | 0.92 |
|  | Wingless | 0.36 | -0.44 | -0.13 | 0.14 | 0.21 | 0.21 | 0.08 | 0.21 | 0.17 | 0.2 | 0.14 | 0.20 | 0.11 | -0.05 | 0.14 |
|  | 28S | 0.00 | 0.00 | 0.00 | 0.00 | 0.00 | 0.00 | 0.00 | 0.00 | 0.00 | 0.00 | 0.00 | 0.00 | 0.00 | 0.00 | 0.00 |

Table S5 *F*_ST_ values among populations of each *Leistus* and *Nebria* species

* DX (Daxueshan) population is found only in *L. nokoensis* complex and G (Guanyuan) population is found only in *N. uenoiana*

|  | Genes | X/N | X/H | X/Y | N/H | H/Y | N/Y | DX/X*  G /X* | DX/H* G/H* | DX/N* G/N* | DX/Y*  G/Y* | T/G | X/T | H/T | N/T | Y/T |
| --- | --- | --- | --- | --- | --- | --- | --- | --- | --- | --- | --- | --- | --- | --- | --- | --- |
| *L. nokoensis* complex | COI | 0.006 | 0.009 | 0.048 | 0.008 | 0.049 | 0.050 | 0.036 | 0.035 | 0.038 | 0.041 | - | - | - | - | - |
|  | 16S | 0.000 | 0.000 | 0.006 | 0.000 | 0.006 | 0.006 | 0.004 | 0.004 | 0.004 | 0.006 | - | - | - | - | - |
|  | Wingless | 0.000 | 0.001 | 0.007 | 0.001 | 0.008 | 0.007 | 0.001 | 0.002 | 0.001 | 0.008 | - | - | - | - | - |
|  | 28S | 0.001 | 0.001 | 0.006 | 0.000 | 0.005 | 0.005 | 0.000 | 0.001 | 0.001 | 0.006 | - | - | - | - | - |
| *L. smetanai* | COI | 0.030 | - | - | - | - | - | - | - | - | - | - | - | - | - | - |
|  | 16S | 0.005 | - | - | - | - | - | - | - | - | - | - | - | - | - | - |
|  | Wingless | 0.008 | - | - | - | - | - | - | - | - | - | - | - | - | - | - |
|  | 28S | 0.001 | - | - | - | - | - | - | - | - | - | - | - | - | - | - |
| *N. niitakana* | COI | 0.054 | - | - | - | - | - | - | - | - | - | - | - | - | - | - |
|  | 16S | 0.006 | - | - | - | - | - | - | - | - | - | - | - | - | - | - |
|  | Wingless | 0.011 | - | - | - | - | - | - | - | - | - | - | - | - | - | - |
|  | 28S | 0.001 | - | - | - | - | - | - | - | - | - | - | - | - | - | - |
| *N. formosana* | COI | - | 0.048 | 0.045 | - | 0.018 | - | - | - | - | - | - | - | - | - | - |
|  | 16S | - | 0.008 | 0.012 | - | 0.008 | - | - | - | - | - | - | - | - | - | - |
|  | Wingless | - | 0.005 | 0.010 | - | 0.005 | - | - | - | - | - | - | - | - | - | - |
|  | 28S | - | 0.004 | 0.002 | - | 0.002 | - | - | - | - | - | - | - | - | - | - |
| *N. uenoiana* | COI | 0.003 | 0.004 | 0.005 | 0.004 | 0.005 | 0.003 | 0.004 | 0.003 | 0.003 | 0.006 | 0.004 | 0.004 | 0.004 | 0.004 | 0.006 |
|  | 16S | 0.000 | 0.000 | 0.000 | 0.000 | 0.000 | 0.000 | 0.000 | 0.000 | 0.000 | 0.000 | 0.002 | 0.002 | 0.002 | 0.002 | 0.002 |
|  | Wingless | 0.004 | 0.002 | 0.005 | 0.004 | 0.006 | 0.005 | 0.005 | 0.005 | 0.005 | 0.006 | 0.005 | 0.004 | 0.004 | 0.003 | 0.005 |
|  | 28S | 0.000 | 0.000 | 0.000 | 0.000 | 0.000 | 0.000 | 0.000 | 0.000 | 0.000 | 0.000 | 0.000 | 0.000 | 0.000 | 0.000 | 0.000 |

Table S6 Proportional divergences among populations of each *Leistus* and *Nebria* species

* DX (Daxueshan) population is found only in *L. nokoensis* complex, and G (Guanyuan) population is found only in *N. uenoiana*
